# Development and validation of an immune infiltration/tumor proliferation-related Notch3 nomogram for predicting survival in patients with primary glioblastoma

**DOI:** 10.3389/fgene.2023.1148126

**Published:** 2023-05-10

**Authors:** Zong-Qing Zheng, Guo-Guo Zhang, Gui-Qiang Yuan, Jia-Hui Hao, Qian-Qian Nie, Ming-Cheng Zheng, Zhong Wang

**Affiliations:** ^1^ Department of Neurosurgery, The First Affiliated Hospital of Soochow University, Suzhou, Jiangsu, China; ^2^ Department of Neurosurgery, Beijing Tiantan Hospital, Capital Medical University, Beijing, China; ^3^ Department of Neurology, The First Affiliated Hospital of Soochow University, Suzhou, Jiangsu, China; ^4^ Department of Neurosurgery, The Fifth Hospital of Xiamen, Xiamen, Fujian, China

**Keywords:** notch receptor, glioblastoma, nomogram, immune-cell infiltration, prognosis

## Abstract

**Background:** Notch receptors (Notch 1/2/3/4), the critical effectors of the Notch pathway, participate in the tumorigenesis and progression of many malignancies. However, the clinical roles of Notch receptors in primary glioblastoma (GBM) have not been fully elucidated.

**Methods:** The genetic alteration-related prognostic values of Notch receptors were determined in the GBM dataset from The Cancer Genome Atlas (TCGA). Two GBM datasets from TCGA and Chinese Glioma Genome Atlas (CGGA) were used to explore the differential expression between Notch receptors and IDH mutation status, and GBM subtypes. The biological functions of Notch Receptors were explored by Gene Ontology and KEGG analysis. The expression and prognostic significance of Notch receptors were determined in the TCGA and CGGA datasets and further validated in a clinical GBM cohort by immunostaining. A Notch3-based nomogram/predictive risk model was constructed in the TCGA dataset and validated in the CGGA dataset. The model performance was evaluated by receiver operating curves, calibration curves, and decision curve analyses. The Notch3-related phenotypes were analyzed via CancerSEA and TIMER. The proliferative role of Notch3 in GBM was validated in U251/U87 glioma cells by Western blot and immunostaining.

**Results:** Notch receptors with genetic alterations were associated with poor survival of GBM patients. Notch receptors were all upregulated in GBM of TCGA and CGGA databases and closely related to the regulation of transcription, protein-lysine N-methyltransferase activity, lysine N-methyltransferase activity, and focal adhesion. Notch receptors were associated with Classical, Mesenchymal, and Proneural subtypes. Notch1 and Notch3 were closely correlated with IDH mutation status and G-CIMP subtype. Notch receptors displayed the differential expression at the protein level and Notch3 showed a prognostic significance in a clinical GBM cohort. Notch3 presented an independent prognostic role for primary GBM (IDH1 mutant/wildtype). A Notch3-based predictive risk model presented favorable accuracy, reliability, and net benefits for predicting the survival of GBM patients (IDH1 mutant/wildtype and IDH1 wildtype). Notch3 was closely related to immune infiltration (macrophages, CD4^+^ T cells, and dendritic cells) and tumor proliferation.

**Conclusion:** Notch3-based nomogram served as a practical tool for anticipating the survival of GBM patients, which was related to immune-cell infiltration and tumor proliferation.

## 1 Introduction

Glioma is the most common of all primary central nervous system tumors. It was graded from Ⅰ to Ⅳ depending on the tumor’s malignant status (Louis et al., 2021a). Glioblastoma (GBM) is the major subtype of grade Ⅳ glioma and one of the deadliest cancers, with only a 3% 5-year survival rate (Ostrom et al., 2013). Surgical resection, radiotherapy, and chemotherapy are the main methods for glioblastoma therapy. Despite advances in treatment, disease recurrence usually occurs within 6 months, and patients generally die of disease progression in a little over a year (Stupp et al., 2005). Therefore, it is urgent to explore effective tools to predict the survival of patients with GBM. Even though some prognostic biomarkers have been widely validated for predicting GBM survival, these indicators cannot fully elucidate the individual variants and benefit clinical practice well.

Notch signaling is highly conserved in humans and is involved in regulating a variety of cellular processes throughout life, including cell proliferation, stem cell maintenance, cell fate decisions, and differentiation (Aster et al., 2017a). It has been identified that there are four Notch receptors (Notch receptors, Notch1–4) and five types of Notch binding ligands (Delta-like-1, -3, and -4 (DLL-1, 3, 4) and Jagged-1, and -2 (JAG-1, -2) in mammals. After Notch receptors bind to their ligands, Notch intracellular domain (NICD) is transported to the nucleus and regulates the expression of downstream genes, which contributes to the determination of cell fate (Zhou et al., 2022). The dysregulation of Notch signaling is confirmed in multiple cancers and is closely related to cancer progression (Aster et al., 2017b). Particularly in glioma, the Notch pathway participates in tumor development and growth, as well as cancer invasion and recurrence. This is due to the roles of aberrant activation of Notch components in GBM, such as overexpression of DLL-4 and JAG-1 were detected in GBM endothelial cells and promoted the maintenance and differentiation of glioma stem cells via activating the down-stream Hes1 in tumor cells (Zheng et al., 2021). Furthermore, high expression of DLL-4 has been shown to be correlated with glioma angiogenesis (Nandhu et al., 2014), and Notch activation via gp130/STAT3 signaling confers resistance to chemoradiotherapy (Koerdel et al., 2021). Although the Notch pathway has been confirmed to play a critical role in glioma progression *in vitro* and *in vivo*, the values and application of Notch receptors in clinical practice for primary GBM have not yet been completely elucidated.

In this study, we comprehensively explored the expression pattern and biological functions of each Notch receptor in GBM at gene and mRNA levels using TCGA and CGGA databases and validated their expression at protein level in clinical GBM samples. Moreover, the relationships between the Notch receptors and GBM subtypes were further explored to test their application for clinical classification. We also performed survival analysis to screen out the independent clinicopathologic factors with Kaplan-Meier and Cox regression analyses. A novel nomogram and prognostic risk model were constructed and validated in GBM cohorts (IDH1 mutant/wild type and IDH1 wild type) from TCGA and CGGA databases. Finally, we examined the role of Notch3 expression in tumor immune infiltration and tumor cell proliferation.

## 2 Materials and methods

### 2.1 Ethics statement and GBM samples

70 primary GBM samples with complete clinical follow-up data were collected from the department of neurosurgery of the First Affiliated Hospital of Soochow University and Sanbo Brain Hospital of Capital Medical University from 2013 to 2015 (Table S1). All the patients undergo standard therapy treatment of maximum surgical resection combined with radio/chemotherapy. Informed consent was obtained from the GBM patients for the use of brain tissue and for access to medical records for research purposes. The brain tumors were confirmed by neurosurgeons, radiologists, and pathologists based on a physical examination, neuroimaging, and histological examination. The method was in accordance with the World Medical Association’s Declaration of Helsinki. The study was approved by the Ethics Committee of the First Affiliated Hospital of Soochow University. The experimental protocols were also supported by the Ethics Committee of the First Affiliated Hospital of Soochow University.

### 2.2 TCGA and CGGA GBM databases

WHO IV primary GBM samples (IDH1-wild type and IDH1 mutant) from The Cancer Genome Atlas (TCGA) and Chinese Glioma Genome Atlas (CGGA) databases were used in this study. The mRNA sequencing data of Notch receptors and clinical patients’ information were downloaded and tidied from GlioVis (http://gliovis.bioinfo.cnio.es/) (Bowman et al., 2017) and the official CGGA website (www.cgga.org.cn) (Zhao et al., 2021). Then we explored the prognostic and diagnostic values of Notch receptors in GBM, including differential expression analysis, survival analysis, and correlation analysis between Notch receptors and GBM subtypes. The mRNA expression z-scores relative to diploid samples were set at 1.5. The data was analyzed by SPSS software and the online bioinformatic analysis tools (https://www.xiantao.love/) by R (V3.6.3).

### 2.3 Genetic alteration analysis of notch receptors

cBioPortal (http://www.cbioportal.org/) was widely used for genetic alteration analysis based on the TCGA database (Gao et al., 2013). We included 592 GBM samples (Pan Cancers Atlas, 378 cases with complete mutation and CNA data) to analyze the different genetic alterations type of Notch receptors in GBM. Moreover, we also explored the relationship between the genetic alteration of Notch receptors and the prognosis of GBM patients with Kaplan-Meier survival analysis.

### 2.4 Biological function analysis of notch receptors

We screened out the top 50 genes that were most similar to each Notch receptor in GBM from GEPIA (http://gepia2.cancer-pku.cn/) (Tang et al., 2017) (Supplementary File S1). The protein-protein interaction network was performed using the STRING database (https://string-db.org) (Szklarczyk et al., 2019) (Supplementary Figure S1). We performed and visualized the analysis of Gene Ontology (GO) and Kyoto Encyclopedia of Genes and Genomes (KEGG) by Cytoscape software.

### 2.5 Immunostaining

Paraffin-embedded tissue sections were dewaxed (xylene, graded ethanol), peroxidase activity quenched (0.3% hydrogen peroxide), and antigen-retrieved; The U87 and U251 glioma cells were fixed in 4% paraformaldehyde. Then they were further blocked with 5% goat serum incubated with primary antibodies (Notch1:100, Abcam, ab52301; Notch2: 1:50, Santa Cruz Biotechnology, sc-518169; Notch3 1:50, Santa Cruz Biotechnology, sc-518169; Notch4 1:50 dilutions, Santa Cruz Biotechnology, sc-32613, Ki67, abcam15580) at 4°C overnight. Subsequently, specific secondary antibodies were used to incubate the sections or glioma cells for 1 hour at 37°C. Then the sections were immersed in ABC peroxidase with diaminobenzidine (Beyotime, China) and counterstained with Mayer hematoxylin for 2 min (Beyotime, China). The U87 and U251 glioma cells were stained and covered with a DAPI solution. A microscope (Nikon, Tokyo, Japan) was used to photograph the staining signals. ImageJ Pro (Media Cybernetics, Rockville, Maryland, United States) was used by two observers (blinded to the experimental groupings) for statistical analysis.

### 2.6 Evaluation of immunostaining

All GBM tissue sections were evaluated by two independent observers. The semi-quantitative evaluation for Notch receptors expression was referred to in the previous studies (Dell'Albani et al., 2014). The percentage of positive cells in the five random areas was assessed and scored under a 400x light microscope. The frequency of Notch1–4 staining was evaluated on a scale of 0–4 (0: <5%, 1: 5%–25%, 2: 25%–50%, 3 50%–75%, 4:>75%). The expression of Notch receptors scored≤1 was the low expression, while Notch receptors scored >1 was the high expression. All areas of the specimen were examined. The score presented the predominant cell staining intensity in each case.

### 2.7 Cell culture with Notch3 knockdown

Human glioma cell lines (U87MG, U251) were purchased from the American Type Culture Collection (Virginia, USA). The U87 and U251 glioma cell lines were incubated in 10% FBS DMEM mediums. A lentiviral packaging kit was purchased from GeneChem (Shanghai, China) to generate stable Notch3-knockdown (Notch3 shRNA) glioma cell lines. According to the manufacturer’s protocol, before we began the knockdown, the U251 and U87 were thawed and incubated at a concentration of 5*10^5^ per well (six-well plate) overnight. The next day we changed the medium and added lentiviral vectors that encoded Notch3 small hairpin RNA (shNotch3) and nontargeting shRNA (shNT) to the medium for a 3-day infection. Finally, the U251 and U87 cell lines were harvested for protein extraction. Western blot was used to detect the knockdown efficiency of Notch3. Cell proliferation was determined by Ki-67staining. The primers for shNotch3 and shNT are provided in Supplementary Materials.

### 2.8 Survival analysis, model construction and evaluation

By Kaplan-Meier method with a log-rank test, we analyzed the relationship between Notch receptors and overall survival (OS) of GBM patients (IDH1-wild type and IDH1 mutant) in TCGA, CGGA, and our clinical GBM cohort. Univariate and Multivariate Cox analysis was applied to analyze the influence of Notch3 expression on GBM prognosis along with other clinicopathologic factors (Age, Gender, IDH mutation status, chemotherapy status, radiotherapy status). The Median of the Notch3 expression level was used as the cut-off value. In all tests, *p* < 0.05 was defined as statistically significant. The TCGA GBM database was set as a training cohort (IDH1-wild type and IDH1 mutant, *n* = 247, Supplementary Table S2), and the CGGA GBM database was used for an external validation cohort (IDH1-wild type and IDH1 mutant, *n* = 190, Supplementary Table S3). Acquired from multivariate Cox analysis, we used the independent prognostic factors to construct a nomogram and predictive risk score model to assess the GBM prognosis for 1, 2, and 3 years, respectively. The following equation was utilized to compute the predictive risk score: Y = 0.488*Notch3+0.472*age-0.998*idh-0.810*chemo-2.237*radio. The TCGA GBM database (IDH1-wild type, *n* = 229) and the CGGA GBM (IDH1-wild type, *n* = 159) were used to test the performance of the predictive risk score in IDH1-wild type GBM. To determine the accuracy, reliability, and clinical benefits of the predictive risk model, time-dependent receiver operating curves (ROC), calibration curves, and decision curve analysis were utilized, respectively. All the data were analyzed and visualized using the online bioinformatic analysis tools (https://www.xiantao.love/) by R (V3.6.3).

### 2.9 Single-cell analysis and functional state analysis

GBM single-cell sequencing data (GSE57872) were used to explore the correlation between Notch3 expression and the functional state of glioma cells. All the data were analyzed and visualized on Cancer SEA (http://biocc.hrbmu.edu.cn/CancerSEA/home.jsp) (Yuan et al., 2019). The workflow has been summarized in Supplementary Figure S2.

### 2.10 Statistical methods

The data were statistically analyzed and visualized using GraphPad Prism 8 (GraphPad, San Diego, CA, United States). Data from two groups were analyzed by Student’s unpaired two-sided *t*-test. Other statistical comparisons between more than two groups were performed using one-way ANOVA and *post hoc* least significant difference tests for multiple comparisons. R (V3.6.3) and SPSS software (version 24.0, IBM, SPSS, Chicago, IL) was used to perform Kaplan–Meier survival analyses with log-rank tests. Cox proportional hazards regression was used for the multivariate analysis. Receiver operating curves (ROC) analysis was performed using probabilities to calculate the area under the curve (AUC). *p* < 0.05 was considered statistically significant for all statistical analyses.

## 3 Results

### 3.1 The alteration of notch receptors in GBM at the genetic level

To determine the genetic alteration of Notch receptors in GBM, we included 592 GBM samples with 378 mutation and CNA data in TCGA (Pan Cancers Atlas). The genetic alteration rates of Notch receptors were analyzed via cBioPortal. A total alteration rate (25/378,6.6%) of Notch receptors was detected in the GBM patients, and the alteration rate of Notch receptors ranged from 1.9% to 2.9%.

**FIGURE 1:**
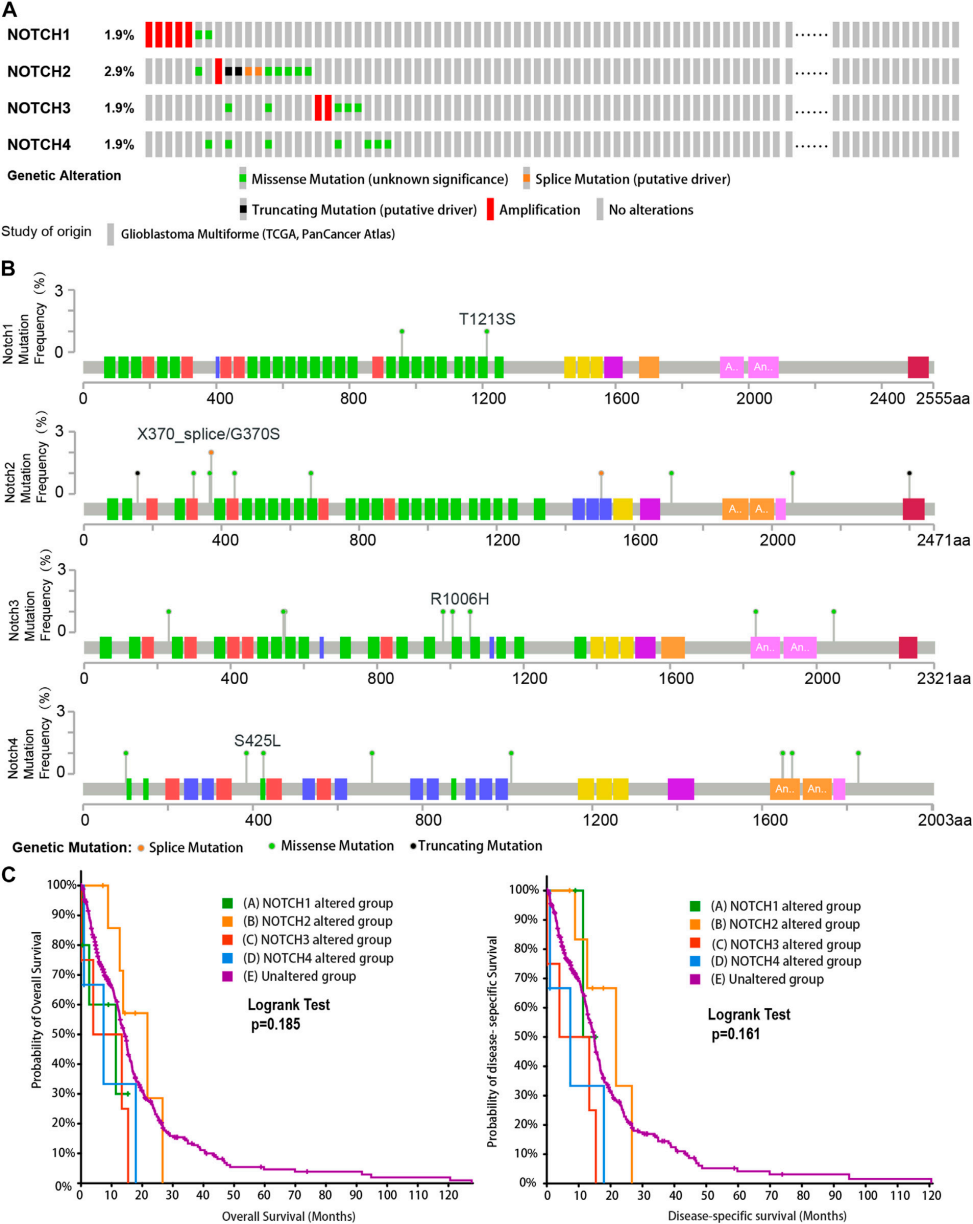
The genetic alteration and clinical value of Notch receptors in the GBM cohort from TCGA database. **(A)** The rates and types of genetic alteration of Notch receptors in the GBM dataset from TCGA (Pan Cancers Atlas, *n* = 378). **(B)** The genetic mutation frequency and mutation sites of Notch receptors in the GBM dataset from TCGA. **(C)** Kaplan–Meier plots for overall survival (OS) and disease-specific survival (DSS) in GBM patients with Notch receptor alteration and un-alteration. *p*-values were calculated from log-rank tests and *p* < 0.05 was considered statistically significant.

As is shown in Figure 1A,7 (1.9%) GBM patients presented Notch1 genetic alterations, including missense mutation and amplification. Notch2 showed the highest rate of genetic alteration, and 11 (2.9%) GBM cases indicated missense mutation, splice mutation, truncating mutation, and amplification. Notch3 displayed the same types of genetic alterations as Notch1. There are 7 (1.9%) GBM samples that displayed missense mutation and amplification in Notch3. Only 7 GBM samples (1.9%) presented missense mutation in Notch4. Additionally, the mutation frequencies in different sites of notch receptors were close to 1% and most types of gene mutations in Notch receptors (like missense mutation) were sparsely distributed from 0 to 2000 sites of the amino acid (Figure 1B).

To further explore the clinical value of genetic alteration of each Notch receptor in GBM, we performed the corresponding survival analysis. We found that GBM patients with the genetic alteration of Notch1 and Notch4 have a shorter median overall survival and median disease-specific survival than the unaltered group, especially Notch3 presented the shortest median overall survival and median disease-specific survival (Figure 1C), suggesting an essential role of Notch receptors in the GBM progression.

### 3.2 The expression patterns and biological roles of notch receptors in GBM at the mRNA level

**FIGURE 2:**
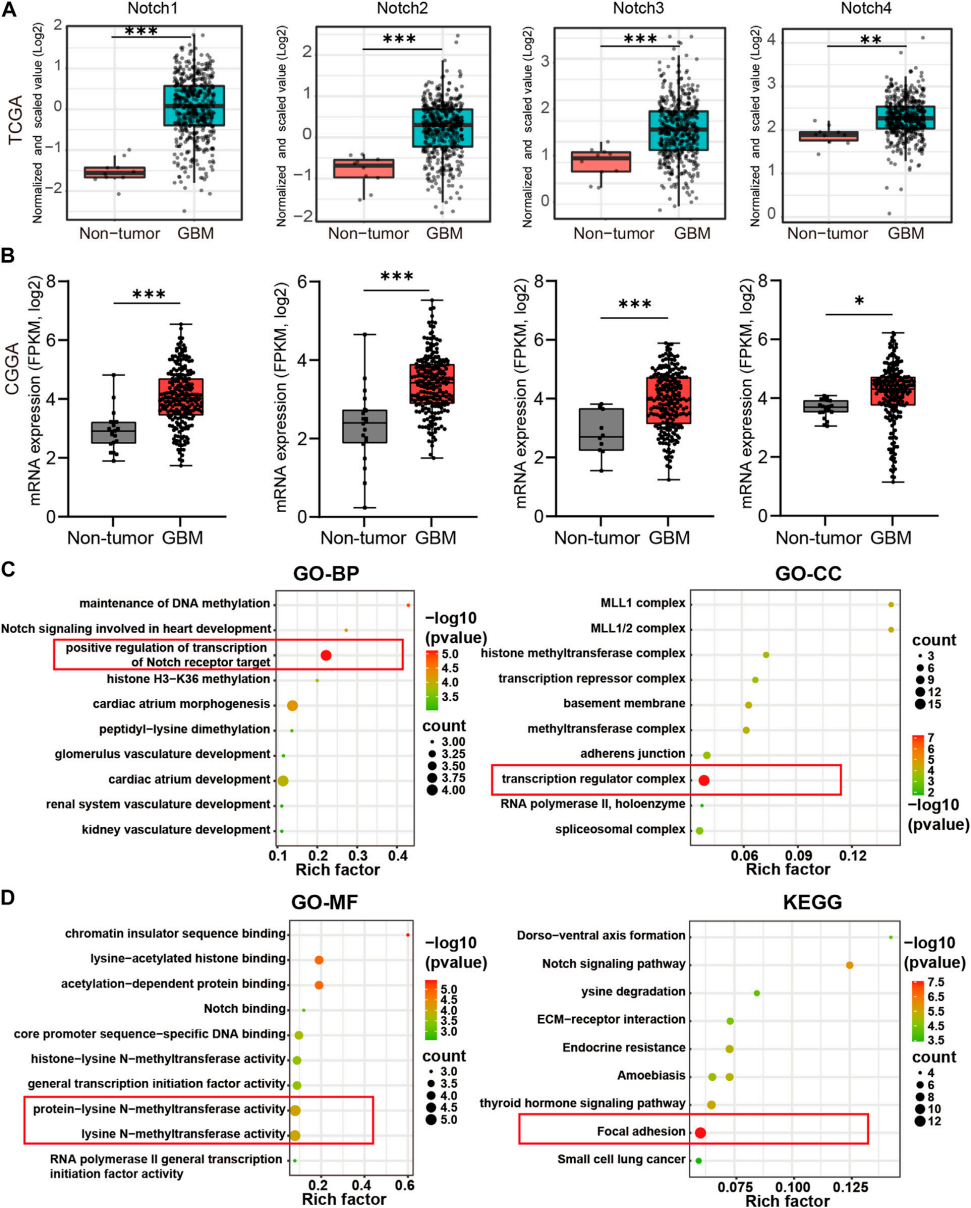
The differential expression and biological functions of Notch receptors in GBM from TCGA and CGGA database. **(A)** The expression of Notch receptors at mRNA level in GBM and normal tissues from TCGA database. **(B)** The expression of Notch receptors at mRNA level in GBM and normal tissues from CGGA database, *p < 0.05, **p < 0.01, ***p < 0.005, ns represents no significance. **(C, D)** The functional enrichment of Notch receptors with their top 50 similar genes in GBM by GO and KEGG analysis, including biological process (BP), cellular component (CC), molecular function (MF), and KEGG pathway. The red box represented the best functional enrichment score.

To further examine the expression variation of Notch receptors at the mRNA level, we downloaded the mRNA-seq sequencing data from TCGA and CGGA databases. The differential expression analysis was performed to evaluate the expression patterns of Notch receptors in GBM. As is shown in Figure 2, All Notch receptors (including Notch1, Notch2, Notch3, and Notch4) presented significantly higher expression at mRNA level in GBM tissues than in normal tissues both in TCGA and CGGA databases (Figures 2A, B), suggesting that all Notch receptors might have oncogenic roles in the tumorigenesis and progression of GBM.

To further explore the oncogenic roles of Notch receptors in GBM, we screened out the top 50 × 4 genes significantly associated with Notch receptors in GBM from TCGA data (Supplementary Material). The protein-protein interaction network showed that nucleic acid binding genes, including U2AF2, HNRNPM, and SETD1A, and transcription factor binding genes, such as CREBBP and SIN3A, were most related to Notch receptors (Supplementary Figure S1). We also performed GO and KEGG analysis to explore the biological functions of the top 50 genes by Cytoscape software. The results of biological process (BP) and cellular component (CC) indicated that Notch receptors were related to the positive regulation of transcription of the Notch receptor target and exerted an effect on the transcription regulator complex, as indicated by the red box (Figure 2C). Moreover, the molecular function (MF) analysis demonstrated that the function of Notch receptors was associated with protein-lysine N-methyltransferase activity and lysine N-methyltransferase activity. Notably, Notch receptors related genes were enriched in focal adhesion of tumor cells except for the classical Notch signaling pathway (Figure 2D). These findings suggested that Notch receptors were related to transcription regulation of the Notch target gene during the malignant GBM progression and expansion.

### 3.3 The relation between notch receptors and IDH mutational status and GBM subtypes

**FIGURE 3:**
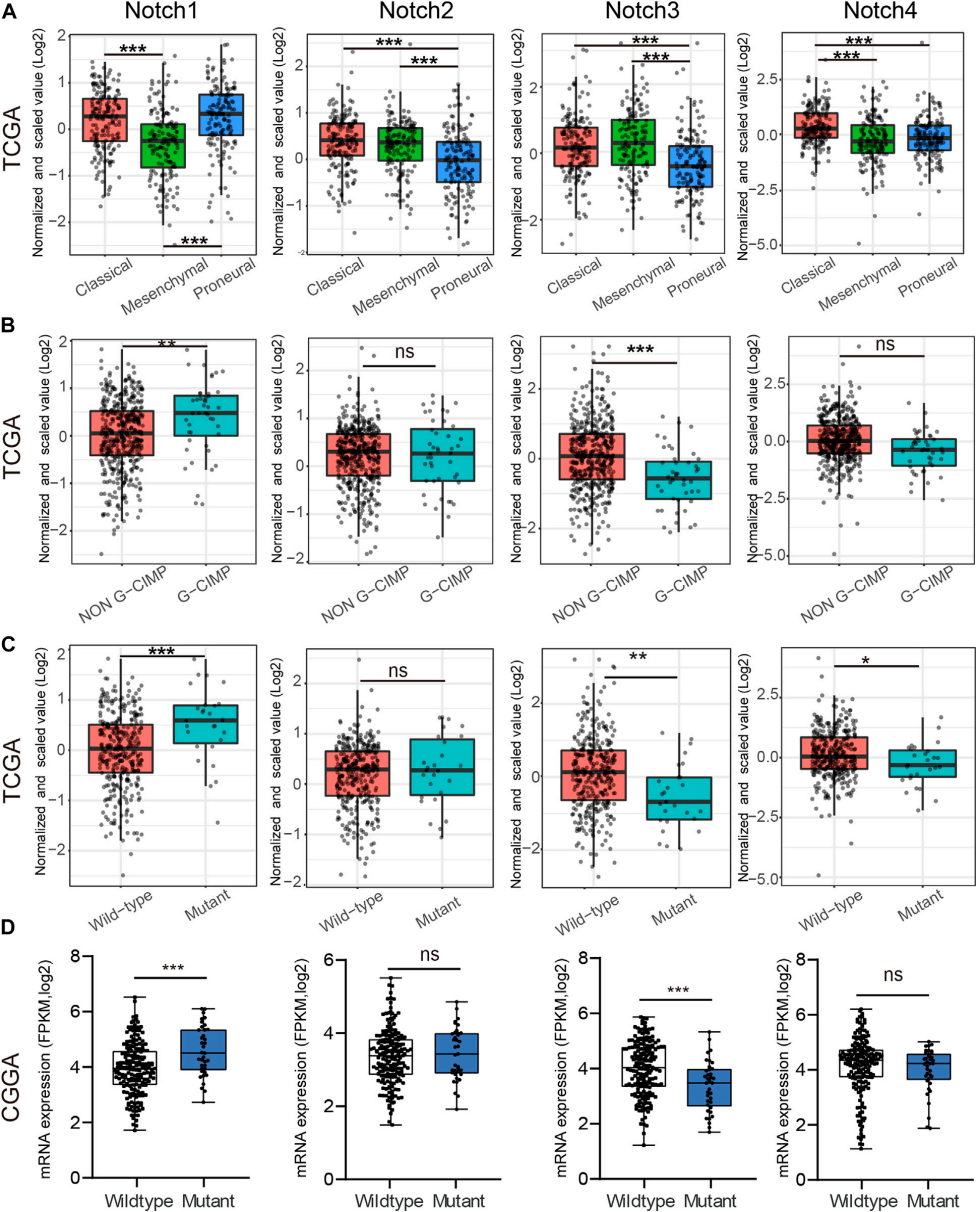
The correlation between Notch receptors expression and IDH mutational status, and GBM subtypes. **(A)** The expression of Notch receptors (Notch 1/2/3/4) in Classical, Mesenchymal, and Proneural subtypes of GBM from the TCGA database. **(B)** The mRNA expression of Notch receptors (Notch 1/2/3/4) in G-CIMP and Non-G-CIMP subtypes of GBM from CGGA database. **(C, D)** The mRNA expression of Notch receptors (Notch 1/2/3/4) in IDH wild-type and IDH mutant GBM from TCGA and CGGA databases. *p < 0.05, **p < 0.01, ***p < 0.005, ns represents no significance.

GBM was divided into three subtypes, including Proneural, Classical, and Mesenchymal types, based on the pathological features, which indicated different survival of GBM patients (Wang et al., 2017). Thus, we tested whether Notch receptors were associated with different GBM subtypes in the TCGA database. As shown in Figure 3A, Notch1 in Classical and Proneural subtypes of GBM showed significantly higher expression than that in the Mesenchymal subtype, while Notch2 and Notch3 displayed high expression in Classical and Mesenchymal subtypes of GBM and low expression in Proneural subtype. Notch4 in GBM showed a notable differential expression between Classical and, Proneural subtypes.

Additionally, CpG Island Methylator Phenotype (G-CIMP) is another type of GBM classification. Patients with G-CIMP tumors are usually younger at the time of diagnosis and experience significantly improved outcomes (Noushmehr et al., 2010). Thus, we examine the possible relationship between Notch receptor expression and G-CIMP types in GBM. As is indicated in Figure 3B, Notch1 and Notch3 showed increased expression in non-G-CIMP subtypes of GBM, suggesting that Notch1 and Notch3 served as important markers for GBM G-CIMP classification.

Isocitrate dehydrogenase (IDH) mutation, a significant event in GBM progression, has been widely used as a diagnostic and prognostic marker for GBM patients (Yan et al., 2009). GBM patients with IDH mutation possess a relatively favorable clinical outcome. Next, we explored the possible relationship between Notch receptors and IDH mutation status in GBM from CGGA and TCGA databases. The TCGA result indicated a higher expression of Notch1 in IDH1-mutant GBM as compared to the IDH1 wild-type GBM. On the contrary, Notch3 and Notch4 showed a higher expression in IDH1 wild-type samples as compared to IDH1-mutant samples (Figure 3C). Interestingly, the Notch receptors analysis from CGGA databases presented similar results to the TCGA database for Notch1 and Notch3 (Figure 3D). Together, these data indicated that Notch1 and Notch3 were correlated with IDH mutation status, suggesting they might act as promising markers for predicting GBM survival and GBM IDH classification.

### 3.4 The expression patterns and prognostic values of notch receptors expression in GBM

**FIGURE 4:**
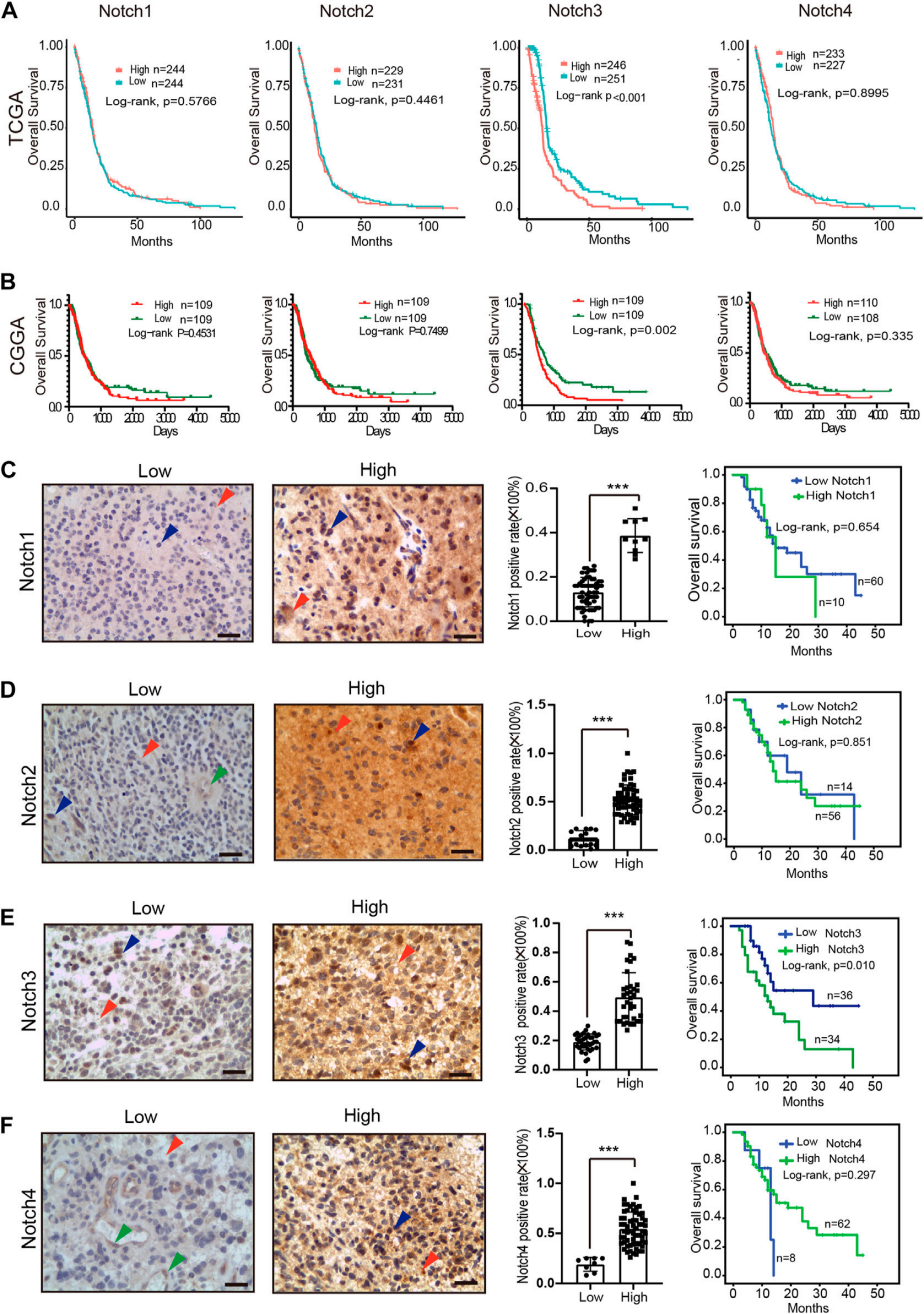
The expression pattern and prognostic value of Notch receptors expression in GBM. **(A)** Kaplan–Meier plot for overall survival in the GBM patients with low- and high-expression of Notch receptors from the TCGA database, *p*-values were calculated from log-rank tests. **(B)** Kaplan–Meier plot for overall survival in the GBM patients with low- and high-expression of Notch receptors from the CGGA database, stratified by median. *p*-values were calculated from log-rank tests. **(C–F)** The expression patterns of Notch receptors at protein level using immunohistochemical staining in clinical GBM samples (left); Kaplan–Meier plot for overall survival in the clinical GBM patients (*n* = 70) with high and low expression of Notch receptors (right). Red arrowhead means positive staining in the cytomembrane of tumor cells, blue arrowhead means positive staining in the nucleus of tumor cells, green arrowhead means positive staining in the endothelial cells, scale bar means 50 μm; *p < 0.05, **p < 0.01, ***p < 0.005.

To determine the prognostic values of Notch receptors in GBM, we conducted a comprehensive Notch receptors survival analysis in GBM patients with clinical follow-up data in TCGA and CGGA databases. We found that GBM patients with high expression of Notch3 displayed a shorter survival time, while the expression of Notch1, Notch2, and Notch4 failed to show a prognostic significance in GBM (Figures 4A, B).

To verify the expression and clinical prognostic values of Notch receptors in GBM at the protein level, we collected 70 cases of GBM samples with complete clinical follow-up data from our medical center. Immunohistochemical staining was used to detect the Notch receptors' expression, and the semi-quantitative assessment method was used to evaluate the levels of Notch receptors' expression. As indicated in Figures 4C–F, positive staining of Notch receptors was mainly displayed in the cytomembrane of tumor cells (red arrowhead) and minorly presented in the nucleus of tumor cells (blue arrowhead) in GBM samples. The mildly positive staining of Notch receptors (Notch2/4) was also observed in the cytomembrane of endothelial cells (green arrowhead). 70 cases were divided into the high expression of Notch receptors and low expression of Notch receptors based on the Notch receptors' semi-quantitative score. The result indicated that 14% (10/70) of GBM cases presented a high expression of Notch1. Furthermore, over half of all cases displayed a high expression of Notch2 (80%, 56/70), Notch3 (51.4%, 36/70), and Notch4 (88.5%, 62/70). These data indicated that a large number of GBM samples presented a high expression of Notch receptors at the protein level, which was parallel to their expression at mRNA levels, suggesting a critical role of Notch receptors expression in the GBM initiation and progression.

Similar to the mRNA expression-based survival analysis, we also performed a Kaplan-Meier analysis based on the protein expression levels of Notch receptors in GBM tissue. The results demonstrated that GBM patients with high expression of Notch3 at protein levels presented with a poor prognosis, while the expression of Notch1, Notch2, and Notch4 showed no relation to GBM patient survival, which was consistent with the results in TCGA and CGGA at mRNA levels (Figures 4C–F). Together, these data demonstrate that Notch3 may present a prognostic implication for GBM patients.

### 3.5 Construction of a Notch3-based nomogram and risk score model to predict the GBM patients’ survival

**FIGURE 5:**
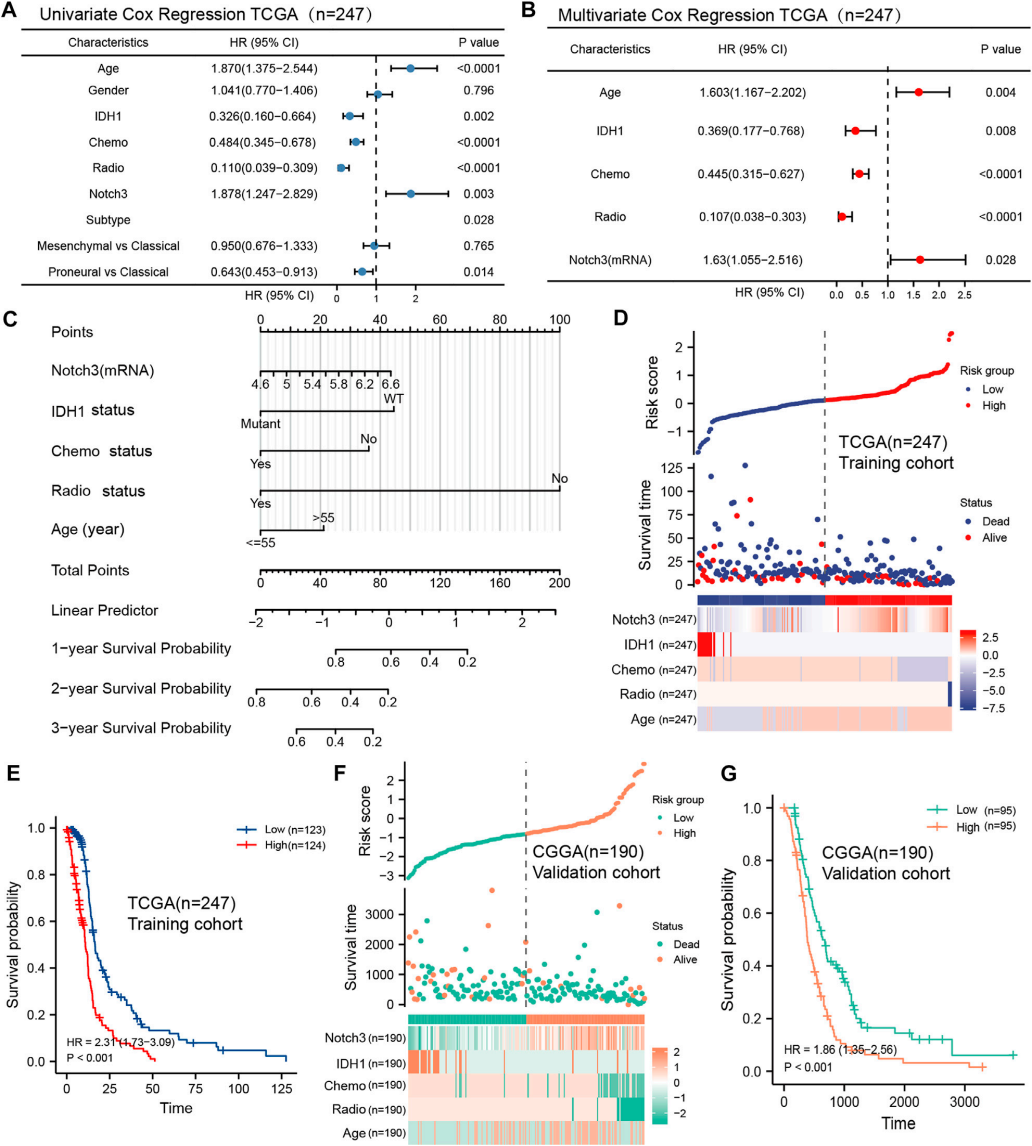
Construction of a Notch3-based nomogram and risk score model to predict the GBM patients’ survival. **(A, B)** Forest plots showing univariate and multivariate Cox proportional hazard ratios with 95% confidence intervals and *p*-values for age, gender, IDH1, Chemo, Radio, Notch3, and GBM subtypes in the TCGA database. **(C)** A nomogram constructed by the independent prognostic clinicopathologic factors (Notch3, IDH1 status, chemo status, radio status, age) to predict the probability of 1-, 2-, and 3-year survival of GBM patients. **(D)** A Notch3-based prognostic risk score model calculated by the independent prognostic clinicopathologic factors (Notch3, IDH1 status, chemo status, radio status, age) for risk stratification in TCGA GBM patients (*n* = 247). **(E)** Kaplan–Meier plot for overall survival in TCGA GBM patients with low- (*n* = 123) and high-risk scores (*n* = 124), stratified by median. *p*-values were calculated from log-rank tests. **(F)** The Notch3-based prognostic risk score model for risk stratification in CGGA GBM patients, *n* = 190. **(G)** Kaplan–Meier plot for overall survival in CGGA GBM patients with low- (*n* = 95) and high-risk scores (*n* = 95), stratified by median. *p*-values were calculated from log-rank tests.

To further confirm the independent prognostic role of Notch3 in primary GBM, we included age, gender, IDH mutation status, GBM subtypes, chemotherapy status, radiotherapy status, and Notch3 expression to perform Cox survival regression analysis in the TCGA database. The univariate analysis results indicated that age, IDH mutation, Notch3 expression, GBM subtypes, chemotherapy status, and radiotherapy status were significant for GBM patients’ survival (Figure 5A). These variables were further included to perform the multivariate analysis. The results indicated that Notch3 showed an independent prognostic value for GBM patients. GBM patients with high Notch3 expression demonstrated a higher risk for poor survival than that with low Notch3 expression (HR = 1.63, *p* < 0.028, Figure 5B).

Except for the Notch3 expression, the age, IDH mutation status, chemotherapy status, and radiotherapy status also presented independent prognostic values for GBM patients’ survival. Thus, we further developed a practical nomogram constructed by these independent clinicopathologic variables to predict 1-, 2-, and 3-year survival probability for the GBM patients (Figure 5C). The point scale of this nomogram was used to assign points to each variable based on the results of multivariate Cox regression. With the adjusted range from 1 to 100, total scores were calculated by adding up the points of each variable. By delineating a direct line down from the total score line to the outcome line, the survival probabilities of each GBM patient at 1-, 2-, and 3- year were defined.

To further explore the prognostic values of Notch3 and other independent clinicopathologic factors for GBM patients’ survival, we selected these variables to construct a prognostic risk score model (PRSM) based on the analysis of multivariate Cox regression (forward likelihood ratio). The TCGA data were used as the training cohort, and the risk score of each GBM patient was calculated by combining the coefficient weighting score of each variable. We divided the TCGA GBM population into high- and low-risk score groups (Figure 5D). Kaplan-Meier survival analysis demonstrates that the GBM patients with high-risk scores presented short survival (HR = 2.31, *p* < 0.001) (Figure 5E).

To verify the prognostic performance of the Notch3-based risk score model in the different populations, we included the CGGA as the external validation cohort and used the same formula to calculate the risk score for each CGGA patient (Figure 5F). Kaplan-Meier survival analysis presented a similar result as those observed for TCGA GBM patients (Figure 5G), suggesting that the Notch3-based risk score model may function as a valuable tool for predicting the GBM patients’ survival.

### 3.6 Evaluation of Notch3-based risk score model in primary GBM and primary IDH1-wild type (WT) GBM

**FIGURE 6:**
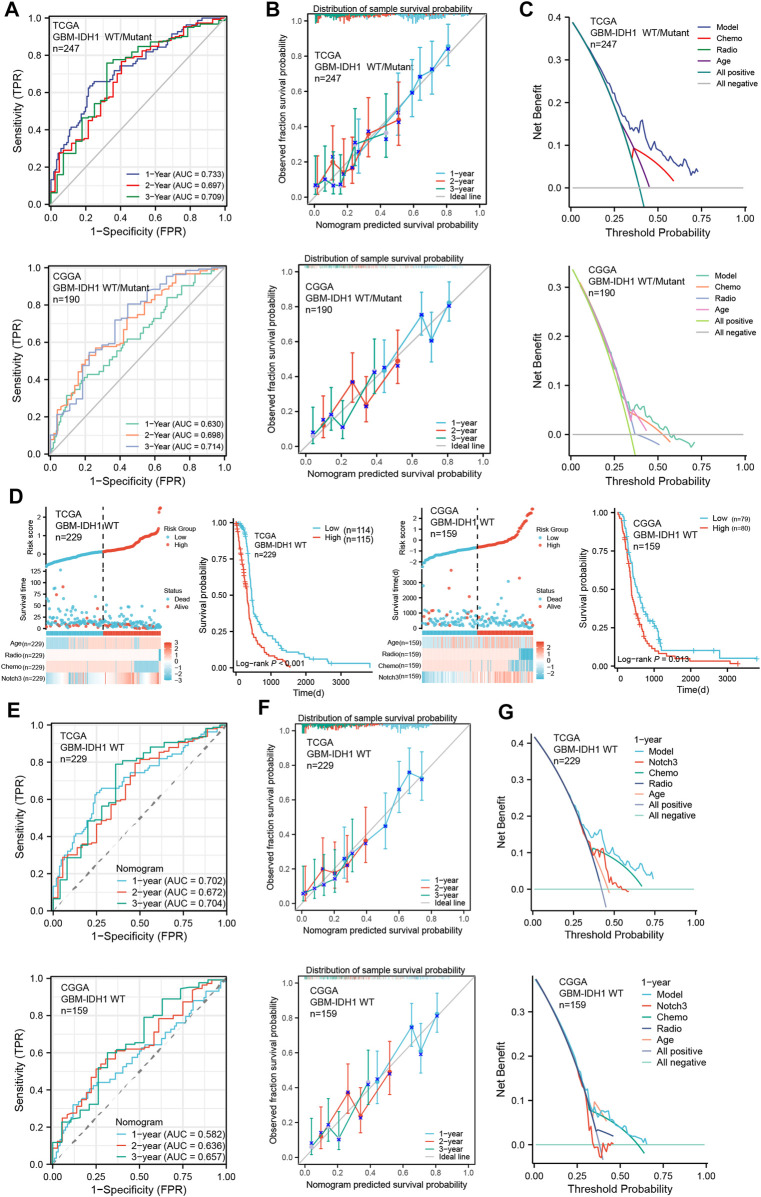
Evaluation of Notch3-based risk score model in primary GBM cohorts. **(A)** Time-dependent receiver operator characteristic curve (t-ROC) for assessing the predictive accuracy of the Notch3-based prognostic risk score model in GBM patients from TCGA and CGGA databases. The predictive accuracy was calculated by 1-,2-, and 3-year AUC. **(B)** The calibration curve for evaluating the consistency between the predictive probability and observative probability of the Notch3-based prognostic risk score model in GBM patients from TCGA and CGGA databases. The consistency was assessed by comparing the ideal line with 1-, 2-, and 3-year predictive lines. **(C)** The decision curve analysis (DCA) for evaluating the net benefits of the Notch3-based prognostic risk score model in the TCGA and CGGA databases. **(D)** The Notch3-based prognostic risk score model for risk stratification in IDH1-WT GBM patients from TCGA and CGGA databases. Kaplan–Meier plot for overall survival in the IDH1-WT GBM patients with high- and low-risk scores from the TCGA and CGGA databases, stratified by median. *p*-values were calculated from log-rank tests. **(E)** T-ROC for assessing the predictive accuracy of Notch3-based prognostic risk score model in the IDH1-WT GBM patients from TCGA and CGGA databases. The predictive accuracy was calculated by 1-, 2-, and 3-year AUC. **(F)** The calibration curve for evaluating the consistency between the predictive probability and observative probability of the Notch3-based prognostic risk score model in the IDH1-WT GBM patients from TCGA and CGGA databases. The consistency was assessed by comparing the ideal line with 1-, 2-, and 3-year predictive lines. **(G)** The DCA for evaluating the net benefits of the Notch3-based prognostic risk score model in the IDH1-WT GBM patients from TCGA and CGGA databases.

To further assess the predictive accuracy of the Notch3-based prognostic risk score model (PRSM) for primary GBM patients’ survival, we calculated the area under the curve (AUC) using analysis of the time-dependent receiver operator characteristic curve (t-ROC). As shown in Figure 6 A, the AUC values of PRSM in the training TCGA cohort and validating CGGA cohort both exceeded 0.6, indicating that the Notch3-based prognostic risk score model (PRSM) was moderately accurate.

Moreover, to assess the authenticity of the Notch3-based prognostic risk score model (PRSM), the calibration curve analysis was performed and demonstrated that survival probability at 1-,2-, and 3- year presented an optimal consistency between the prediction and observation both in the training and external validation cohorts (Figure 6B). Additionally, we also evaluated the clinical benefits of PRSM using the decision curve analysis (DCA). The results demonstrated that Notch3-based PRSM could achieve more net benefits compared with other prognostic variables for almost all threshold probabilities in both the training and validation cohorts (Figure 6C), suggesting that Notch3-based PRSM showed a better-predictive performance for primary GBM patient’s survival.

According to the 2021 WHO classification, only GBM with IDH1 wild type (WT) was defined as GBM (Louis et al., 2021b). To assess the performance of our Notch3-based PRSM in the IDH1-WT GBM, we excluded the previous GBM with IDH1 mutant from the GBM datasets in TCGA and CGGA and assessed the IDH1-WT GBM using Notch3-based PRSM. As is indicated by Kaplan-Meier survival analysis, the IDH1-WT GBM patients with high-risk scores presented shorter survival than that with high-risk scores (Figure 6D). Moreover, the analyses of t-ROC, calibration curve, and DCA indicated a good performance of Notch3-based PRSM in predicting IDH1-WT GBM patients’ survival (Figures 6E–G). These data suggested that our Notch3-based PRSM presented a stable value in predicting the GBM patients’ survival.

### 3.7 Notch3-related immune infiltrates

**FIGURE 7:**
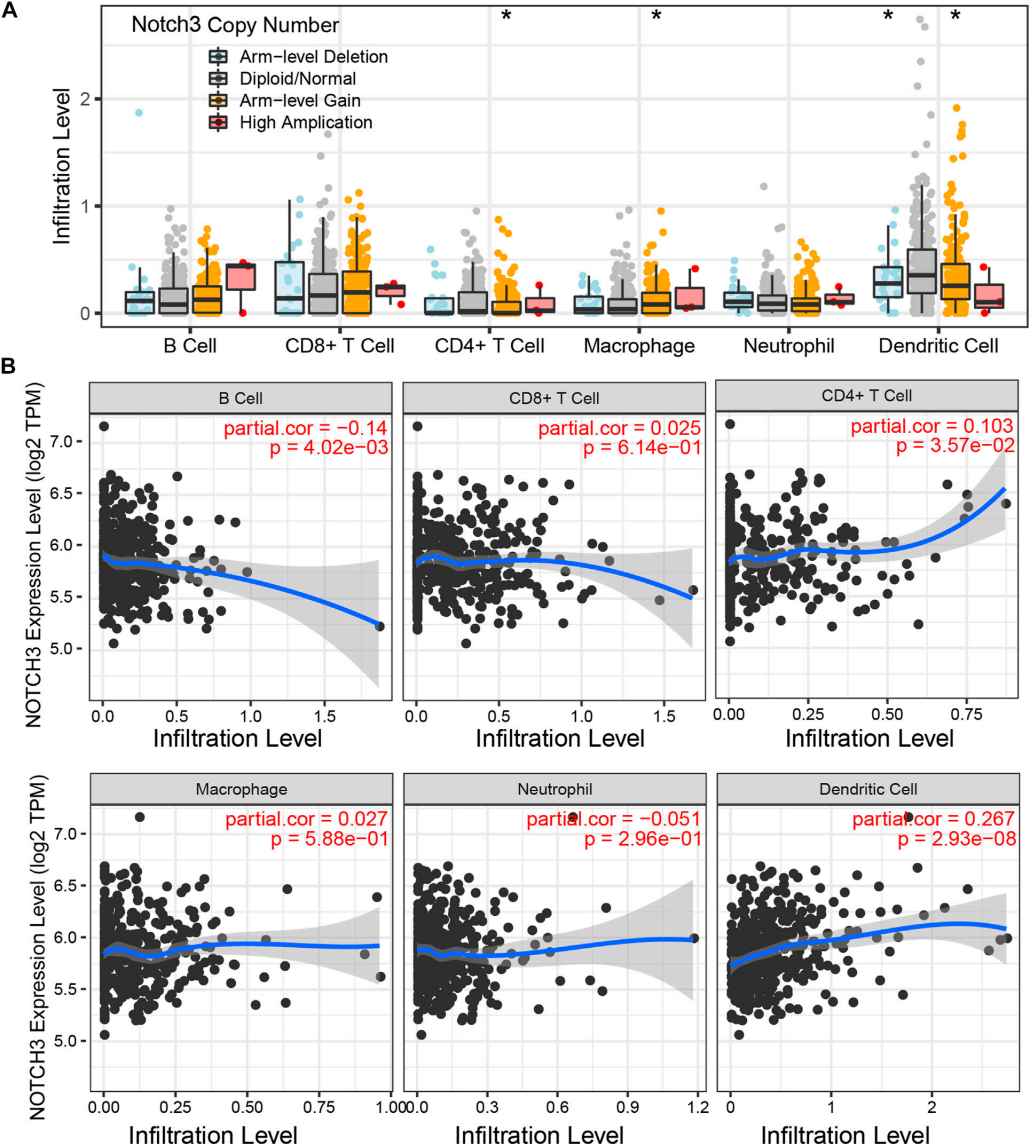
Notch3-related immune infiltrates. **(A)** The relation between Notch3 copy number alterations and the infiltration level of various immune cells (B cell, CD8^+^ T cell, CD4^+^ T cell, Macrophage, Neutrophil, and Dendritic cell). **(B)** The relation between Notch3 mRNA expression and the infiltration level of various immune cells (B cell, CD8^+^ T cell, CD4^+^ T cell, Macrophage, Neutrophil, and Dendritic cell). *p < 0.05, **p < 0.01, ***p < 0.005.

As immune-cell therapy has a positive effect on GBM clinical outcomes (Sokratous et al., 2017), we aimed to explore the correlation between Notch3 and various immune cells. Based on the TIMER, we found that arm-level gain alteration of Notch3 was significantly associated with immunological of macrophages, CD4^+^ T cells, and Dendritic cells (Figure 7A); while on the mRNA levels, Notch3 expression is negatively related to B cells and positively correlated to the CD4^+^ T cells and dendritic cells (Figure 7B), which implies that Notch3 present a promising potential in assessing the efficacy of GBM immunotherapy.

### 3.8 Single-cell analysis and validation of the correlation between Notch3 and tumor proliferation

**FIGURE 8:**
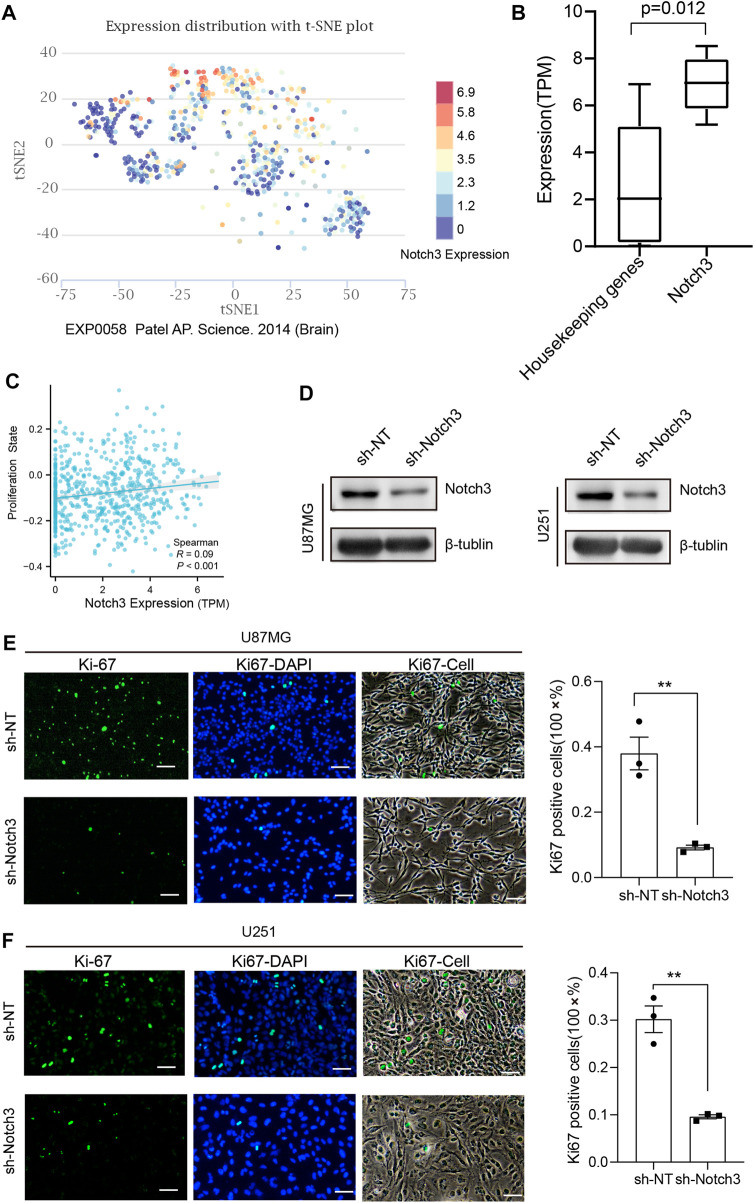
Notch3 expression is closely related to tumor proliferation in GBM. **(A)** The differential expression distribution of Notch3 in GBM sample using single-cell analysis. **(B)** The expression range of Notch3 and housekeeping genes in different GBM cells. **(C)** The correlation between Notch3 and tumor proliferation state, Spearman R = 0.09, *p* < 0.01. **(D)** Notch3 knockdown efficiency in U251 and U87 glioma cells was assessed by Western blot, and β-tubulin was set as a loading control. **(E, F)** The proliferation state of U251 and U87 glioma cells with knockdown of Notch3 was assessed by Ki-67 immunostaining. scale bar = 50 μm; all the data were presented as means ± SD; *p < 0.05; **p < 0.01; ***p < 0.005.

To explore the effect of Notch3 expression on the functional state of GBM, we next performed the single-cell analysis in the primary GBM dataset (GSE57872) and used CancerSEA (http://biocc.hrbmu.edu.cn/CancerSEA/home.jsp) to analyze and visualize the results. The distribution and range of Notch3 expression are shown in Figures 8A, B. We explored the possible relationship between notch3 expression and GBM phenotypes. We found that Notch3 expression is positively related to tumor proliferation (Figure 8C). To validate this relationship, we knocked down the Notch3 expression in GBM cell lines (U87MG and U251) to detect the alteration of cell proliferation by Ki-67 staining. The results demonstrated that Notch3 knockdown significantly reduced the propagation of glioma cells (Figures 8D–F), suggesting that Notch3-mediated GBM progression is closely related to tumor cell proliferation.

## 4 Discussion

Notch receptors, the critical components of Notch signaling pathways, have been confirmed to participate in tumor initiation, progression, and recurrence (Aster et al., 2017b). However, the clinical values and application of Notch receptors for primary GBM have not yet been completely elucidated. In this study, we comprehensively analyzed the changes in mRNA expression and structural variation of Notch receptors using TCGA and CGGA databases and further confirmed the protein expression and clinical value in our clinical GBM database. We found that the Notch receptor with genetic alteration in GBM was correlated with poor prognosis. All the Notch receptors presented high expression at the mRNA level in GBM tissue. We pinpointed that high expression of Notch3 showed an independent prognostic value in GBM. Based on the Cox regression results, we constructed and validated a novel Notch3-based nomogram for predicting the survival of patients with primary GBM. Finally, we confirmed the role of Notch3 in tumor immune infiltration and tumor proliferation.

The expression of Notch receptors and their clinical roles in GBM remains controversial among various reports. Previous studies have shown that Notch1 displayed a high expression in GBM (El Hindy et al., 2013) and was correlated with the patient’s poor prognosis (Li et al., 2011; Hai et al., 2018). This is related to the promoting effects of Notch1 in the phenotype of glioma stem cells (Wang et al., 2019; Yi et al., 2019). Furthermore, high expression of Notch1 has been observed in Classical and Proneural Subtypes of GBM (Hai et al., 2018), which was in line with our results, suggesting a critical role of Notch1 in these two GBM subtypes. Whereas in our study, Notch1 both presented a high expression in TCGA and CGGA, but it showed no relation to GBM patient survival, which may be ascribed to the different number of GBM samples in the study.

Notch2 has been confirmed to play an oncogenic role in many malignancies (Xiu and Liu, 2019). However, the exact role of Notch2 in GBM has not been reported. In particular, Notch2 was reported to display a weak expression at the protein level in 11 cases of GBM samples (Dell'Albani et al., 2014), which is different from our results that over half of GBM samples (80%, 56/70) presented with a high expression of Notch2. Moreover, the high expression of Notch2 in our study was closely related to the Classical subtype of GBM and showed no association with IDH1 mutation status. These data were partly consistent with previous results that Notch signaling is highly expressed in the Classical subtype (Verhaak et al., 2010) Additionally, our results further demonstrated that the expression of Notch2 at mRNA or protein levels showed no relationship with patients’ prognosis, suggesting that Notch2 may play a minor role in the GBM initiation and progression.

Notch 3 was also reported to be activated in glioma and played a significant role in glioma cell proliferation, cell migration, invasion, and apoptosis based on the *in vitro* experiment (Alqudah et al., 2013). As GBM is the highest grade and most malignant tumor in glioma, our results were consistent with the partial phenotype of previous results. Based on the GBM single-cell analysis, we confirmed that notch3 expression was positively correlated with tumor proliferation in primary GBM samples and *in vitro* experiments. Furthermore, Notch3 expression was closely associated with poor-prognosis-related GBM subtypes, including IDH1 wild-type, Classical and Mesenchymal subtypes, suggesting that Notch3 may be a promising marker for GBM prognosis.

Moreover, we performed the Kaplan-Meier survival analysis of Notch3 expression within TCGA and CGGA databases and validated its expression and prognostic value at protein levels in our GBM cohort. The results demonstrated that GBM patients with high expression of Notch3 indicated a shorter survival period, which was similar to the previous reports (Shen et al., 2015) that Notch3 gene polymorphism is associated with the prognosis of gliomas. Additionally, we also confirmed the independent prognostic roles of Notch3 in two primary GBM cohorts with univariate and multivariate Cox regression analysis, suggesting that notch3 may serve as a useful biomarker for primary GBM prognosis.

To further explore the clinical value and application of notch3 in primary GBM, we constructed a novel nomogram using Notch3 expression and other independent clinicopathological factors based on the Cox regression analysis, which could serve as a practical tool for predicting the survival of GBM patients with considering individual variation. Furthermore, based on these independent prognostic factors, we developed and validated a prognostic risk model for GBM patients that contributes to assessing their prognosis. Time-dependent ROC curves, calibration curves, and decision curve analysis all presented that our prognostic risk models presented good accuracy and reliability, which might provide clinical benefit in assessing survival in GBM patients. Despite our prognostic risk model being established in the previous GBM cohorts (IDH1 mutant and IDH1-WT), it showed a high performance in predicting the prognosis of GBM patients with IDH-WT that was redefined as GBM in the 2021 WHO classification of CNS tumors (Louis et al., 2021b).

Notch4 signaling not just affects tumor cell biological behaviors but also is responsible for tumor angiogenesis (Xiu et al., 2021). Interestingly, our data demonstrated a similar result that Notch4 showed positive staining both in tumor cells and endothelial cells, suggesting Notch4 may play dual roles respectively in tumor cells and endothelial cells. Despite that Notch 4 presented a differential expression in GBM at mRNA and protein levels. Its expression showed no clinical significance in predicting patients’ survival.

Notwithstanding the practicability of our findings, some limitations in our study need to be addressed. First, despite strict inclusive and exclusive criteria, selection and recall bias are unavoidable because of the retrospective design. Second, although we have included two of the large population of GBM cohorts, the prognostic model could be improved in a larger dataset to ensure its robustness in the future. Third, due to the limitation of molecular pathological diagnosis in our GBM sample and differences in clinicopathological information, some essential molecular features like IDH1 mutation status were not available in 70 clinical samples. Fourth, as we confirmed the relation between notch3 expression and tumor cell proliferation, the potential mechanisms need to be explored in the future. Anyway, this study provided a practical nomogram and prognostic risk model for assessing the survival of patients with GBM based on multi-omics and multi-database analysis.

## Data Availability

The datasets presented in this study can be found in online repositories. The names of the repository/repositories and accession number(s) can be found in the article/Supplementary Material.
