# Supplementary material for: Development and validation of an immune infiltration/tumor proliferation-related Notch3 nomogram for predicting survival in patients with primary glioblastoma
Source: Front Genet. 2023 May 10;14:1148126. doi: 10.3389/fgene.2023.1148126 (PMC10240236; doi:10.3389/fgene.2023.1148126)
Supplement: Supplementary file 1 [file Table1.DOCX]

**Table S1** Clinical pathological characteristics of 70 GBM samples

| Clinical features | Clinical outcome | | | P value |
| --- | --- | --- | --- | --- |
|  | Alive | Dead | |  |
| **Age**  <55years  >55years  **Gender**  Female  Male  **KPS score**  <70  >70 | 18  17  18  17  16  19 | 21  14  12  23  20  15 | 0.470  0.147  0.339 | |
| **Notch 1** |  |  |  | |
| Low | 31 | 29 | 0.734 | |
| High | 4 | 6 |  | |
| **Notch 2** |  |  |  | |
| Low | 6 | 8 | 0.550 | |
| High | 29 | 27 |  | |
| **Notch 3** |  |  |  | |
| Low | 25 | 11 | 0.001 | |
| High | 10 | 24 |  | |
| **Notch 4** |  |  |  | |
| Low | 3 | 5 | 0.710 | |
| High | 32 | 30 |  | |
| **OS** | 11.00(12) | 10.00(8) | 0.287 | |

**Table S2** The relation between Notch3 and clinical features in TCGA GBM cohort (n=247)

| Clinical features | Outcome | | P-value |
| --- | --- | --- | --- |
|  | Alive | Dead |  |
| Age |  |  | 0.141 |
| <=55 | 22 | 63 |  |
| >55 | 29 | 133 |  |
| Gender |  |  | 0.285 |
| Female | 21 | 65 |  |
| Male | 30 | 131 |  |
| IDH mutation status |  |  | <0.001 |
| Wildtype Mutant | 41 | 188 |  |
| Mutant | 10 | 8 |  |
| Chemotherapy status |  |  | 0.931 |
| Untreated | 12 | 45 |  |
| Treated | 39 | 151 |  |
| Radiotherapy status |  |  | 0.584 |
| Untreated | 0 | 4 |  |
| Treated | 51 | 192 |  |
| Notch3 expression |  |  | 0.569 |
| Low | 27 | 95 |  |
| High | 24 | 101 |  |

**Table S3** The relation between Notch3 and clinical features in CGGA GBM cohort (n=190)

| Clinical features | Outcome | | P-value |
| --- | --- | --- | --- |
|  | Alive | Dead |  |
| Age |  |  | 0.026 |
| <=55 | 25 | 86 |  |
| >55 | 8 | 71 |  |
| Gender |  |  | 0.071 |
| Female | 18 | 59 |  |
| Male | 15 | 98 |  |
| IDH mutation status |  |  | 0.017 |
| Wildtype Mutant | 23 | 136 |  |
| Mutant | 10 | 21 |  |
| Chemotherapy status |  |  | 0.490 |
| Untreated | 5 | 32 |  |
| Treated | 28 | 125 |  |
| Radiotherapy status |  |  | 0.239 |
| Untreated | 6 | 17 |  |
| Treated | 27 | 140 |  |
| Notch3 expression |  |  | 0.004 |
| Low | 24 | 71 |  |
| High | 33 | 157 |  |
