# Supplementary figures and images for: Development and validation of an immune infiltration/tumor proliferation-related Notch3 nomogram for predicting survival in patients with primary glioblastoma
Source: Front Genet. 2023 May 10;14:1148126. doi: 10.3389/fgene.2023.1148126 (PMC10240236; doi:10.3389/fgene.2023.1148126)

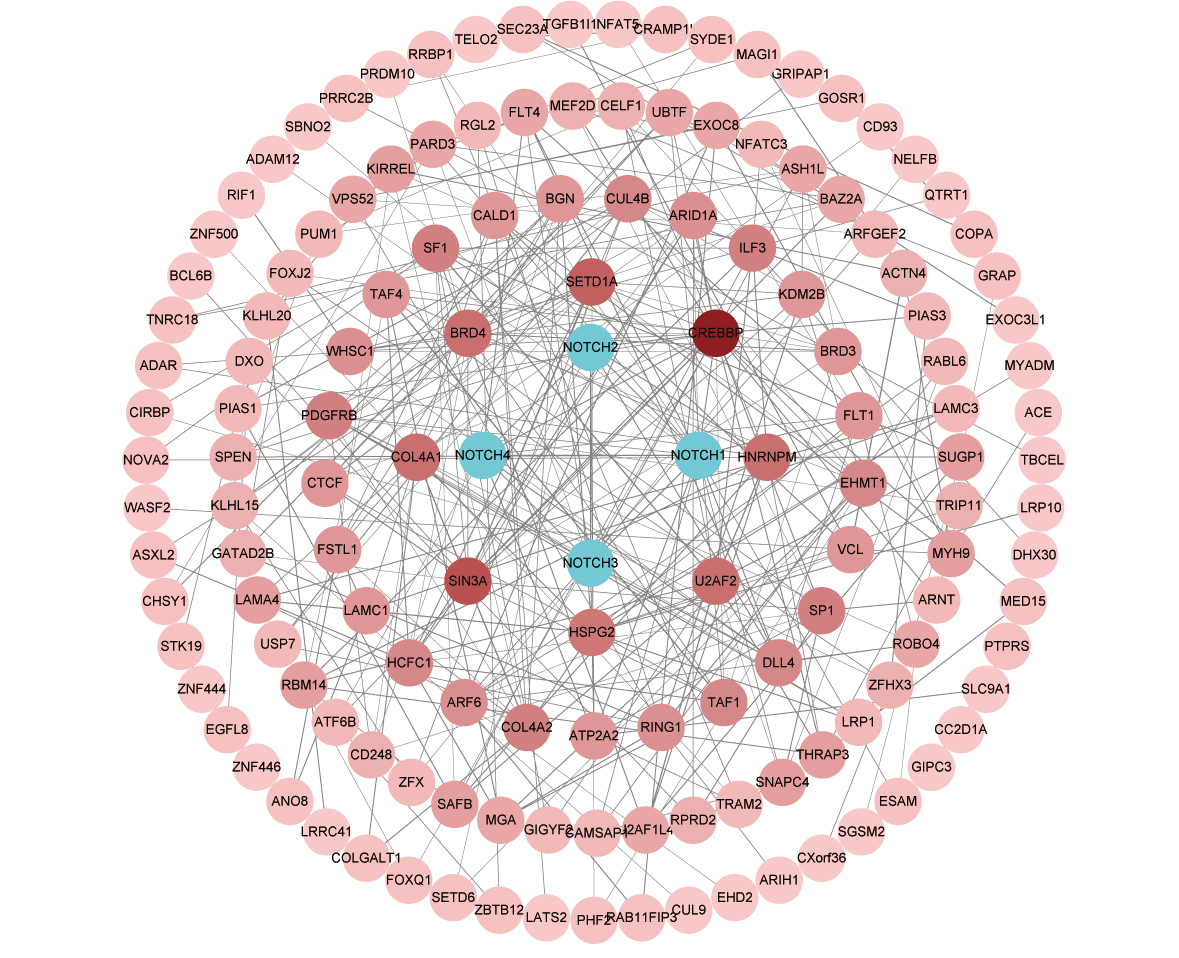

Supplement: Supplementary file 2 [file Image1.JPEG]

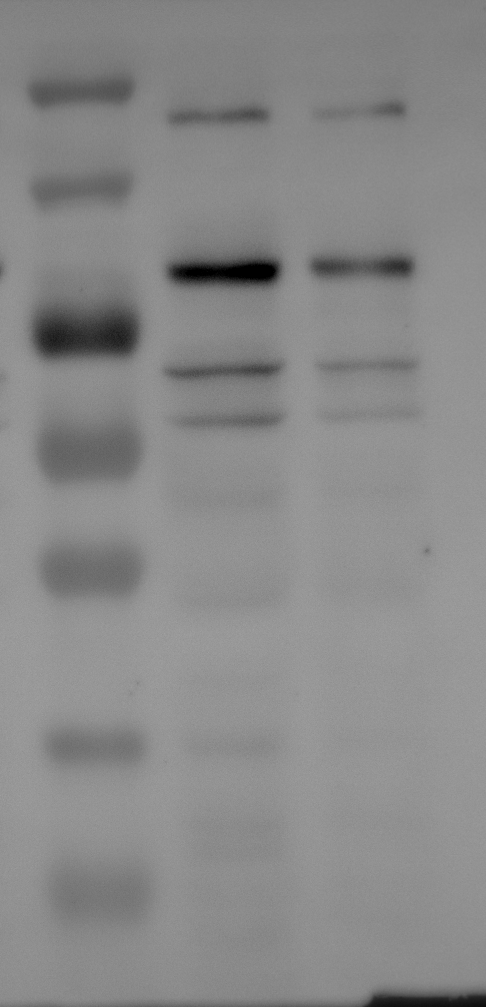

Supplement: Supplementary file 3 [file DataSheet1.ZIP › Western blot origninal figures/U251-Notch3.tif]

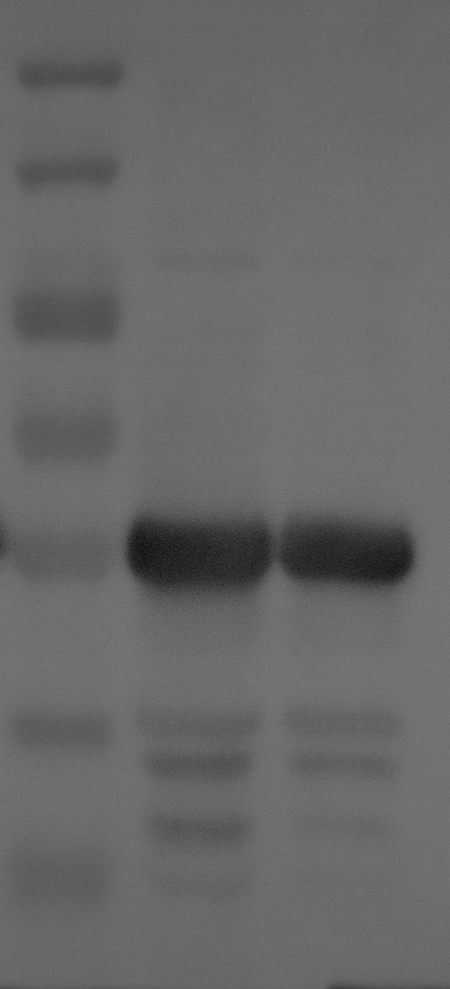

Supplement: Supplementary file 3 [file DataSheet1.ZIP › Western blot origninal figures/U251-β-tubulin.tif]

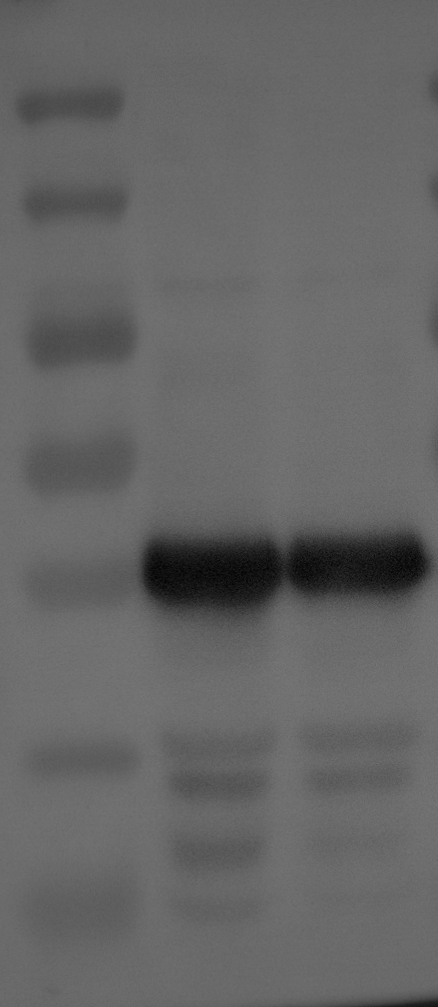

Supplement: Supplementary file 3 [file DataSheet1.ZIP › Western blot origninal figures/U87- β-tubulin.tif]

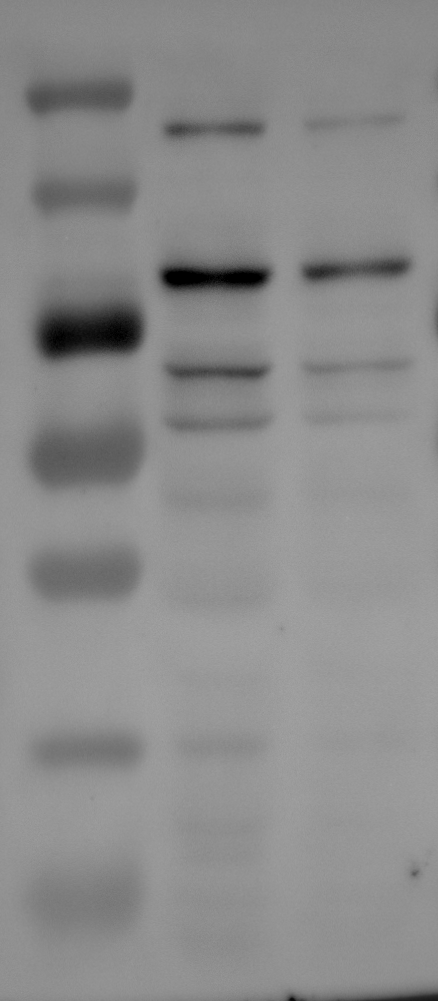

Supplement: Supplementary file 3 [file DataSheet1.ZIP › Western blot origninal figures/U87-Notch3.tif]

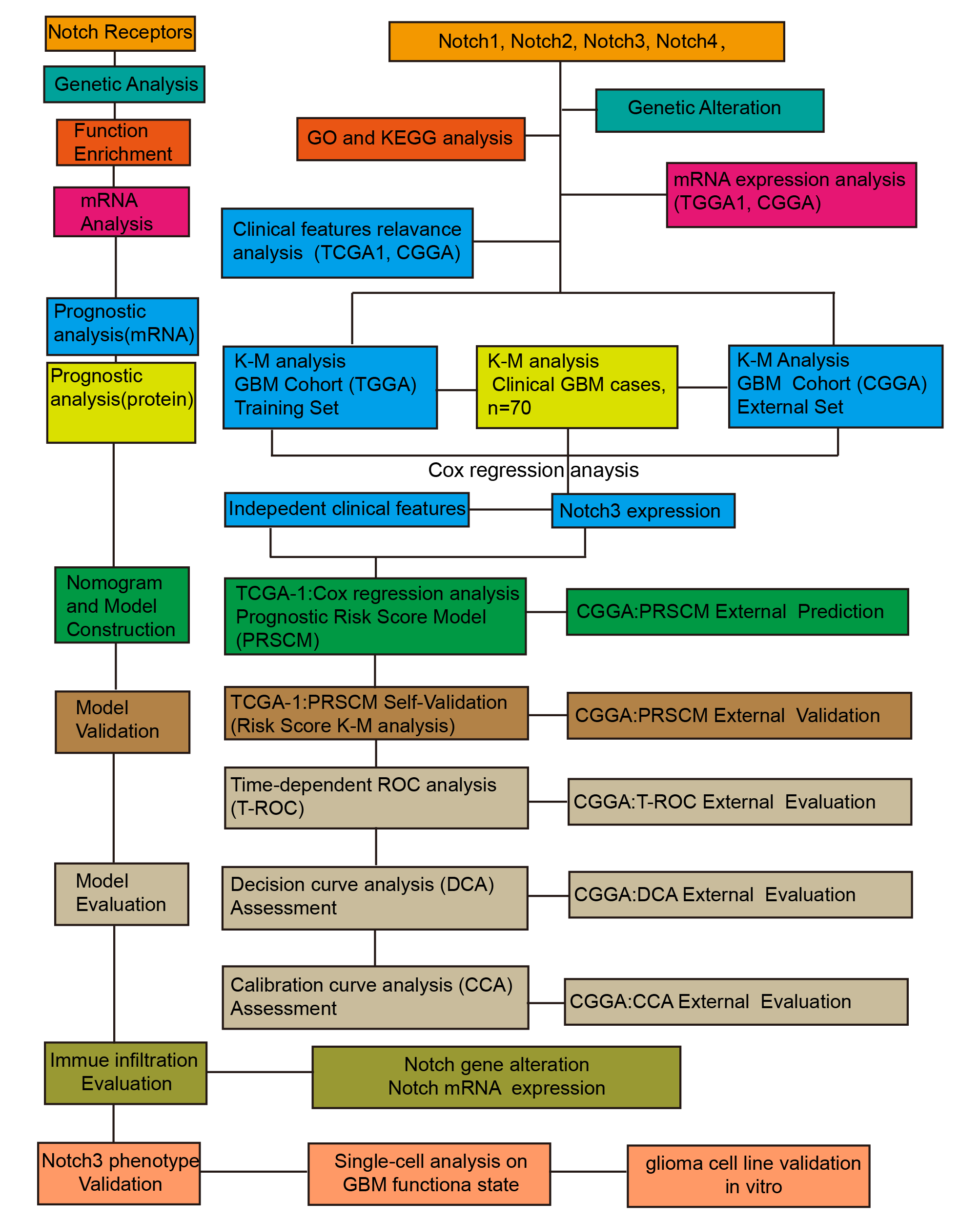

Supplement: Supplementary file 4 [file Image2.TIF]
